# Effects of anthocyanin-rich blackcurrant extract on cardiovascular and metabolic responses during rest and treadmill running: a case study in an elite national level female Southeast Asian endurance athlete

**DOI:** 10.3389/fspor.2026.1818132

**Published:** 2026-07-01

**Authors:** Mark E. T. Willems, Alisa Nana, Poomwut Hiranphan, Sorrawit Wannasorn, Papatsorn Ramyarangsi, Amornpan Ajjimaporn

**Affiliations:** 1 School of Sport, Science and Engineering, University of Chichester, Chichester, United Kingdom; 2 College of Sports Science and Technology, Mahidol University, Nakhon Pathom, Thailand

**Keywords:** anthocyanins, endurance, lactate, marathon, running, substrate oxidation

## Abstract

**Introduction:**

Anthocyanin-rich blackcurrant extract altered exercise-induced metabolic responses (e.g., enhanced fat oxidation) in case studies with Caucasian non-heat acclimatized male endurance athletes. We examined 2–3 weeks before marathon competition the effects of 7-day intake of the extract (i.e., 420 mg of anthocyanins per day) during supine rest and 1 h of moderate-intensity running in an elite national level female Southeast Asian endurance athlete (age: 48 years, BMI: 19.3 kg·m^−2^, body fat: 21.8%, $\dot{V}O_{2\max}$: 44.4 mL·kg^−1^·min^−1^, 28 marathons since 2017) living and training outdoors in Thailand.

**Methods:**

Non-invasive beat-to-beat hemodynamic monitoring (PhysioFlow®) and breath-by-breath gas technology (Cortex Metalyzer 3B) techniques were used during supine rest and a 1-hour indoor (∼24.5 °C, relative humidity: ∼42%) treadmill run at 50% $\dot{V}O_{2\max}$ (7.5 km·h^−1^) with blood lactate (XPER L1) taken 3 min on completion. Study was single-blind and placebo-controlled with final intake of the extract 2 h before starting the treadmill run.

**Results:**

During rest, the blackcurrant extract condition was associated with higher heart rate (+6 bpm), cardiac output (+10%), carbohydrate oxidation (+31%), and respiratory exchange ratio (+0.02 units), with lower systemic vascular resistance (−18%) compared with placebo. Stroke volume, oxygen uptake, and fat oxidation values appeared similar between conditions. During the 1-h treadmill run at 50% $\dot{V}O_{2\max}$, the blackcurrant extract condition was associated with higher stroke volume (+9%), cardiac output (+10%), oxygen uptake (+6%), carbohydrate oxidation (+43%), and respiratory exchange ratio (+0.03 units), with lower systemic vascular resistance (−24%) and fat oxidation (−7%) compared with placebo. Heart rate and rating of perceived exertion values appeared similar between conditions. Post-run lactate concentration was 59% lower in the blackcurrant extract condition (placebo: 1.7 ± 0.1 mMol·L^−1^; extract: 0.7 ± 0 mMol·L^−1^).

**Discussion:**

This case study provided preliminary observations that a highly endurance-trained elite National Southeast Asian female athlete responded physiologically to the intake of blackcurrant extract. Seven-day intake with 420 mg per day of blackcurrant anthocyanins was associated with alterations in cardiovascular, metabolic, and respiratory responses during rest and moderate-intensity running, including lower post-run lactate concentrations. However, the mechanisms underlying this observation remain unclear and should be interpreted cautiously given the exploratory single-participant design.

## 1 Introduction

Polyphenols are naturally occurring compounds that are categorized as flavonoids, phenolic acids, lignans and stilbenes. The blackcurrant berry is rich in two flavonoid anthocyanidins, i.e., delphinidin and cyanidin. Anthocyanidin glucosides (i.e., anthocyanins) are naturally present in many berries as well as in dark-colored fruits and vegetables (1, 2). Some polyphenols, including anthocyanins, may act as dietary antioxidants and influence physiological functions in humans, including vascular vasodilation and modulation of glucose transporters (3–8). Such physiological responses by regular intake of anthocyanins are also known to provide health benefits [e.g., (9, 10)], and may have meaningful applications as well for sport and exercise.

In a cycling cohort study, it was observed that 7-day intake of 300 mg per day of anthocyanin-rich blackcurrant extract (i.e., 105 mg of anthocyanins) altered substrate utilization in endurance-trained male cyclists, e.g., fat oxidation during 10-min of cycling at 65% $\dot{V}O_{2\max}$ was increased by 27% for the cohort (11). Subsequent cohort and case exercise studies in males and females ranging from recreationally active to ultra-endurance trained participants with cycling, walking and running have confirmed that the intake of blackcurrant extract can alter exercise-induced substrate utilization [e.g., (12–14)]. For example, in recreationally active Caucasian males, 7-day intake of blackcurrant extract (210 mg of anthocyanins per day) enhanced fat oxidation during moderate-intensity treadmill walking (13). However, the evidence on blackcurrant extract effects is not consistent. A study in Southeast Asian males showed that a 7-day intake of blackcurrant extract did not alter exercise-induced substrate utilization during moderate-intensity treadmill walking (15). These observations raise the possibility that physiological responsiveness to anthocyanin-rich blackcurrant extract may differ between populations or environmental contexts, although the underlying mechanisms remain unclear. In male Caucasians, intake of anthocyanin-rich blackcurrant extract altered cardiovascular responses during rest in endurance-trained male cyclists [e.g., (16)] and during treadmill walking in recreationally active males [e.g., (17)]. Participants responded with higher cardiac output and lower total peripheral resistance, suggesting a systemic vasodilatory response (16, 17). Interestingly, in contrast to the metabolic responses, cardiovascular responses to blackcurrant extract were reported in both Caucasian and Southeast Asian recreationally active male cohorts during walking (15, 17).

Most studies examining the metabolic, physiological, and cardiovascular effects of blackcurrant extract have involved participants with a Caucasian background living in non-tropical climates [e.g., (11, 17)], with only a few exceptions (15, 18, 19). Ethnicity and geographic location are known to influence gut microbiome composition (20), which plays an important role in the conversion of anthocyanins into bioavailable metabolites (21). In addition, Southeast Asian endurance athletes commonly train outdoors in hot environments and are therefore likely to be heat-acclimatized compared with athletes training in temperate climates. Previous work has also reported differences in exercise-induced metabolic responses in Southeast Asian individuals, potentially related to habitual dietary patterns (22). Heat acclimation, diet and exercise training can all affect the gut microbiome (23, 24), with potential consequences for anthocyanin-induced plasma availability of metabolites. Heat acclimation, habitual diet, and endurance training may therefore influence physiological responses to anthocyanin supplementation; however, the potential contribution of gut microbiome-related mechanisms remains speculative at this point in time and requires further investigation.

Evidence-based supplementation strategies that support exercise performance enhancements and alter physiological and metabolic responses are primarily justified with research in male, mostly Caucasian cohorts (25). Case studies in males, however, on the metabolic effects of blackcurrant extract have confirmed cohort findings. For example, in ultra-endurance male Caucasian athletes, acute intake [420 mg anthocyanins (26)] and 7-day intake [210 mg anthocyanins per day (14)] altered exercise-induced substrate utilization by enhanced fat oxidation. In contrast, a female Caucasian amateur non-acclimatized ultra-endurance athlete did not respond to the intake of 7-day intake (210 mg of anthocyanins per day) a few weeks before competing in the Marathon des Sables (27).

Metabolic and cardiovascular responses to the intake of New Zealand blackcurrant extract have not been examined in Southeast Asian female endurance athletes. Therefore, the aim of the present case study was to examine the metabolic and cardiovascular responses associated with 7-day intake of a high dose of New Zealand blackcurrant extract (i.e., 420 mg anthocyanins) during rest and treadmill running in an elite National-level Southeast Asian female endurance athlete, 3 weeks before marathon competition. The present study was designed as an exploratory physiological case observation in an underrepresented athlete population, intended to generate preliminary data and inform future controlled studies. Findings from this study will provide non-mechanistic observations whether an elite National female Southeast Asian endurance athlete can respond to the intake of blackcurrant extract. The observations will inform future controlled studies on the effects of blackcurrant during rest and exercise in highly trained Southeast Asian endurance athletes.

## 2 Methods

One highly endurance-trained Southeast Asian female marathon runner (age: 48 years, body mass: 52 kg, height: 164 cm, BMI: 19.3 kg·m^−2^, body fat: 21.8%, V̇O_2max_: 44.4 mL·kg^−1^·min^−1^) volunteered for the study. Based on her competitive marathon history, training status, and performance level, the participant was considered elite National based on national Thai running endurance records. The participant had completed multiple marathons since 2017, including competitive marathon performances consistent with elite National age-category endurance running standards (with a personal best of 3 h and 17 min at Boston marathon). The participant had been living and training outdoors in Thailand. Written informed consent was obtained after being informed about the experimental procedures during the three visits (15/08/2024, 22/08/2024, and 29/08/2024), potential risks, and the right to withdraw. The participant completed the Sydney Marathon on 14/09/2024 with a time of <3 h 21 min, placing within the top 8% of female runners in the 45–49 year age category. For the laboratory study, the participant completed a health history questionnaire and reported no conditions or supplement use that could affect the recording of cardiovascular, physiological, and metabolic parameters. Menstrual status, menopausal status, hormonal contraception use, and endocrine-related variables were not formally assessed in the present exploratory case study. Study approval was obtained according to the Research Ethics Policy of the University of Chichester (approval date: 18/07/2024, approval code: 2324_80).

### 2.1 Experimental design

The study had a single-blind, placebo-controlled design with three visits to the air-conditioning controlled (temperature: 24.8 ± 0.4 °C, humidity: 44 ± 3%) Exercise Physiology laboratory at the College of Sport Sciences and Technology and Mahidol University in Thailand. For all visits, the participant was advised to have a light breakfast ∼90 min before arrival in the laboratory, i.e., ∼150 min before running. Details of the testing protocols are described below. In short, in the first visit (15/08/2024, one month before marathon competition), body composition was measured using dual x-ray absorptiometry, followed by a 10-min familiarizing measurement of cardiovascular, respiratory and metabolic responses during rest on treatment table in a supine position. Subsequently, the participant completed an incremental submaximal treadmill running protocol for lactate threshold measurements and an incremental treadmill running protocol to voluntary exhaustion for the measurement of maximum oxygen uptake (see below for details). The data from both running protocols were also used to determine the running speed at 50% of maximum oxygen uptake for the 1 h treadmill run in the placebo (visit 2: 22/08/2024, ∼3 weeks before marathon competition) and New Zealand blackcurrant extract condition (visit 3: 29/08/2024, ∼2 weeks before marathon competition). The order of testing was decided to avoid the required washout and availability of the participant before competition. The participant abstained from strenuous exercise for 48 h before each laboratory visit but was allowed to continue with her habitual marathon training program between visits. Advice was to take no caffeine-containing products in the morning before each morning visit. Dietary intake was recorded for 2 days prior to visit two and the participant was asked to replicate the intake for visit three. On arrival for each visit, measurements of urine specific gravity (MASTER URC-N*α*, ATAGO Co., Ltd., Tokyo, Japan) (placebo: 1.022, NZBC extract: 1.025, both indicating slight dehydration) and blood pressure (Omron SEM-1, OMRON Healthcare Co., Ltd., Kyoto, Japan) were taken [systolic blood pressure: 92 mmHg (placebo); 95 mmHg (NZBC extract), diastolic blood pressure: 57 mmHg (placebo); 50 mmHg (NZBC extract). Both urine specific gravity values indicated slight dehydration at the start of the experimental sessions, which should be considered when interpreting cardiovascular and metabolic responses.

### 2.2 Visit one—preliminary measurements

The main aim of visit one (15/08/2024) was familiarization of the participant with all testing procedures. Body fat was measured from a full body scan using dual x-ray absorptiometry (Lunar iDXA, enCORE software V18, GE Healthcare, Madison, WI, USA). This was followed by a 10-min recording in rest in a supine position of cardiovascular responses (Physioflow®, Bristol, PA, USA) with the participant fitted with skin electrodes (Ambu®, Bluesensor, Malaysia) according to manufacturer guidelines. Respiratory and metabolic responses were simultaneously recorded using a portable breath-by-breath system (Cortex Metalyzer 3B, CORTEX Biophysik GmbH, Leipzig, Germany). Following the measurement in rest, the participant completed the incremental submaximal running test and the incremental running test to exhaustion (see below).

#### 2.2.1 Incremental submaximal running test and incremental running test to exhaustion

For the incremental submaximal running test, an eight-stage protocol was completed on a motorized treadmill Marquette Series 2000 treadmill, USA) with the gradient set at 1% incline (28). The starting speed was 6 km·h^−1^ with stage increments of 1 km·h^−1^. Each stage lasted 4 min with a 2 min passive recovery between each stage. On completion of each stage, a blood sample with the finger prick method was taken for lactate (XPER L1, TaiDoc Technology Corp., New Taipei City, Taiwan). On completion of the incremental submaximal running test, the participant had a passive recovery of 10 min. Subsequently, the incremental running test to exhaustion to determine maximum oxygen uptake was started with a speed of 6 km·h^−1^ and increments of 1 km·h^−1^ every minute until volitional exhaustion. During the incremental submaximal running test and incremental running test to exhaustion, cardiovascular, respiratory and metabolic responses were recorded (data not presented). A blood sample for lactate was taken 3 min after reaching volitional exhaustion and was 6.4 mMol·L^−1^.

### 2.3 Visits two (placebo condition) and three (NZBC extract condition)

For the placebo and NZBC extract condition, the participant was supplied with 7-days supplementation allowing dosing with four capsules per day and advised to consume them in the morning. The placebo capsules had an identical size and matching color as the NZBC extract capsules and contained 300 mg microcrystalline cellulose M102. Each 300 mg NZBC extract capsule (CurraNZ, Health Currancy Ltd, Surrey, United Kingdom) contained 105 mg of anthocyanins, i.e., 35%–50% delphinidin-3-rutinoside, 5%–20% delphinidin-3-glucoside, 30%–45% cyanidin-3-rutinoside, 3%–10% cyanidin-3 glucoside, with the remaining content consisting mainly of natural plant sugars. Composition information was provided by Health Currancy Ltd (United Kingdom). The participant was advised to consume the last four capsules in each condition with a light breakfast ∼90 min before attending the laboratory for the testing sessions. The optimal dosing strategy for alteration of cardiovascular, physiological, and metabolic responses with NZBC extract is not known. One case study in a Caucasian amateur male Ironman athlete used an acute intake of four capsules (i.e., 420 mg of anthocyanins) of New Zealand blackcurrant extract 2 h before testing and alterations in cycling-induced substrate oxidation were observed during 4 h of indoor cycling (26). In addition, previous work in Southeast Asian males employed a 7-day intake of 210 mg of anthocyanins per day (15).

For the 1-h run at 50% of maximum oxygen uptake at a speed of 7.5 km·h^−1^, the treadmill gradient was set at a 1% incline (28). It was not the intention of the study to examine the responses at race pace to align with previous studies that examined the effects with moderate-intensity exercise. In addition, it was agreed with the participant to have a moderate-intensity run that would not interfere with her habitual marathon training program. Cardiovascular, respiratory and metabolic responses were continuously recorded during the 1-h run. The rating of perceived exertion was recorded every 10 min using the 6–20 Borg scale (29). A triplicate blood sample for lactate was taken on completion of the 1-h run.

The participant recorded her dietary intake for 2 days prior to the first experimental condition (i.e., visit 2) and was instructed to replicate the intake for the subsequent experimental visit (i.e., visit 3). The participant was instructed to avoid the consumption of berries, polyphenol-rich nutritional products, and additional dietary supplements during the experimental period. The participant also confirmed that no additional nutritional supplements or medications known to influence cardiovascular or metabolic function were consumed during the experimental period. No biochemical screening for inflammatory status or illness markers was performed before testing sessions. The analysis of the food diaries was done with INMUcal v.4.0 NB.4, with nutritive values for Thai food (Institute of Nutrition, Mahidol University, Thailand) for carbohydrate, fat and protein intake and total energy intake (kcal). Dietary intake is reported in Table 1.

**Table 1:** Dietary intake (record of 48 h before testing) and breakfast on the day of testing.

Macronutrient and total energy intake for 48 h (2-day average)		
Parameter	Placebo	NZBC
Carbohydrate (g)	212 ± 18	236 ± 48
Fat (g)	86 ± 17	56 ± 21
Protein (g)	84 ± 6	85 ± 20
Total energy intake (kcal)	1,960 ± 203	1,791 ± 157

Macronutrients and total energy intake at breakfast		
Parameter	Placebo	NZBC
Carbohydrate (g)	34	54
Fat (g)	4	5
Protein (g)	3	5
Total energy intake (kcal)	180	286

NZBC, New Zealand blackcurrant.

### 2.4 Data calculations and statistical analysis

Rates of whole-body fat and carbohydrate oxidation were calculated with (Equations 1 and 2) from Frayn (30) and the assumption of negligible protein oxidation:

$$\mathrm{Fat}\;\mathrm{oxidation}\;(g\cdot \min^{-1})=\;1.67\times \dot{V}O_{2}-1.67\times \dot{V}CO_{2}$$ (1)

$$\mathrm{Carbohydrate}\;\mathrm{oxidation}\;(g\cdot \min^{-1})=4.55\times \dot{V}CO_{2}-3.21\times \dot{V}O_{2}$$ (2)

Cardiovascular, metabolic, and physiological data are presented descriptively as mean ± SD and 95% confidence intervals calculated from repeated time intervals during rest (five time intervals) and the 1-h treadmill run (six time intervals). Given the exploratory single-participant design, statistical analyses should be interpreted cautiously because repeated measurements within one participant do not represent independent observations. Therefore, the statistical outcomes are presented to provide descriptive context rather than confirmatory inference. A paired two-tailed *t*-test was used for analysis (GraphPad Prism v5 for Windows, GraphPad Software, La Jolla, USA). Significance was accepted at *P* < 0.05. Interpretation of 0.05 > *P* ≤ 0.1 was according to guidelines by Curran-Everett and Benos (31).

## 3 Results

The following observations are presented descriptively and should be interpreted cautiously given the exploratory single-participant design and the repeated within-subject measurements.

### 3.1 Cardiovascular, metabolic and physiological observations during rest

Table 2 presents the cardiovascular, metabolic and physiological data during 10-min in supine rest. During supine rest, the New Zealand blackcurrant extract condition was associated with higher heart rate, cardiac output, minute ventilation, tidal volume, respiratory exchange ratio, and calculated carbohydrate oxidation values compared with placebo. Stroke volume, oxygen uptake, carbon dioxide production, breathing frequency, and fat oxidation values appeared similar between conditions. Heart rate was 6 beats·min^−1^ higher in the New Zealand blackcurrant extract condition [PL: 95% CI (51, 52 beats·min^−1^), NZBC extract: 95% CI (57, 59 beats·min^−1^), *P* < 0.001]. Stroke volume appeared similar between conditions [PL: 95% CI (80, 86 mL), NZBC extract: 95% CI (84, 85 mL), *P* = 0.25], whereas cardiac output was 9.6% higher [PL: 95% CI (4.45, 4.48 L·min^−1^), NZBC extract: 95% CI (4.81, 4.97 L·min^−1^), *P* < 0.001] with lower systemic vascular resistance by 18.0% [PL: 95% CI (1,181, 1,277 dynes·s·cm^−5^), NZBC extract: 95% CI (992, 1,025 dynes·s·cm^−5^), *P* < 0.001], indicative of systemic vasodilation. Breathing frequency was similar between conditions [PL: 95% CI (17, 18 breaths·min^−1^), NZBC extract: 95% CI (17, 19 breaths·min^−1^), *P* = 0.18], whereas tidal volume was 10.9% higher [PL: 95% CI (342, 430 mL), NZBC extract: 95% CI (407, 449 mL), *P* = 0.04] and minute ventilation was 14.6% higher [PL: 95% CI (6.23, 7.35 L·min^−1^), NZBC extract: 95% CI (7.42, 8.12 L·min^−1^), *P* = 0.02]. Oxygen uptake [PL: 95% CI (195, 239 mL·min^−1^), NZBC extract: 95% CI (217, 233 mL·min^−1^), *P* = 0.41] and carbon dioxide production [PL: 95% CI (154, 185 mL·min^−1^), NZBC extract: 95% CI (172, 189 mL·min^−1^), *P* = 0.18] were similar between conditions. The respiratory exchange ratio was 0.02 units higher in the New Zealand blackcurrant extract condition [PL: 95% CI (0.77, 0.80), NZBC extract: 95% CI (0.79, 0.82), *P* = 0.03], accompanied by higher carbohydrate oxidation. Carbohydrate oxidation were 31% higher [PL: 95% CI (0.064, 0.086 g·min^−1^), NZBC extract: 95% CI (0.083, 0.115 g·min^−1^), *P* = 0.01], whereas fat oxidation was similar between conditions [PL: 95% CI (0.068, 0.090 g·min^−1^), NZBC extract: 95% CI (0.070, 0.079 g·min^−1^), *P* = 0.38].

**Table 2:** Cardiovascular, metabolic and physiological data during 10-min of supine rest. .

Parameters	Placebo	NZBC extract
heart rate (beats·min^−1^)	52 ± 0	58 ± 1
stroke volume (mL)	83 ± 3	84 ± 0
cardiac output (L·min^−1^)	4.46 ± 0.01	4.89 ± 0.06
SVR (dynes·s·cm^−5^)	1,229 ± 39	1,008 ± 13
minute ventilation (L·min^−1^)	6.79 ± 0.45	7.78 ± 0.29
breathing frequency (breaths ·min^−1^)	18 ± 0	18 ± 1
tidal volume (mL)	386 ± 35	428 ± 17
oxygen uptake (mL·kg^−1^·min^−1^)	4.17 ± 0.34	4.33 ± 0.13
oxygen uptake (L·min^−1^)	217 ± 18	225 ± 7
carbon dioxide production (mL·min^−1^)	169 ± 13	181 ± 7
RER	0.78 ± 0.01	0.80 ± 0.01
carbohydrate oxidation (g·min^−1^)	0.075 ± 0.008	0.099 ± 0.013
fat oxidation (g·min^−1^)	0.079 ± 0.009	0.074 ± 0.004

NZBC, New Zealand blackcurrant. SVR, systemic vascular resistance. RER, respiratory exchange ratio. Data for each parameter are presented descriptively as mean ± SD from repeated time intervals during rest or the 1-h treadmill run. Statistical outcomes should be interpreted cautiously because repeated measurements within a single participant do not represent independent observations.

### 3.2 Cardiovascular, metabolic and physiological observations during the 1-h treadmill run

**Figure 1:**
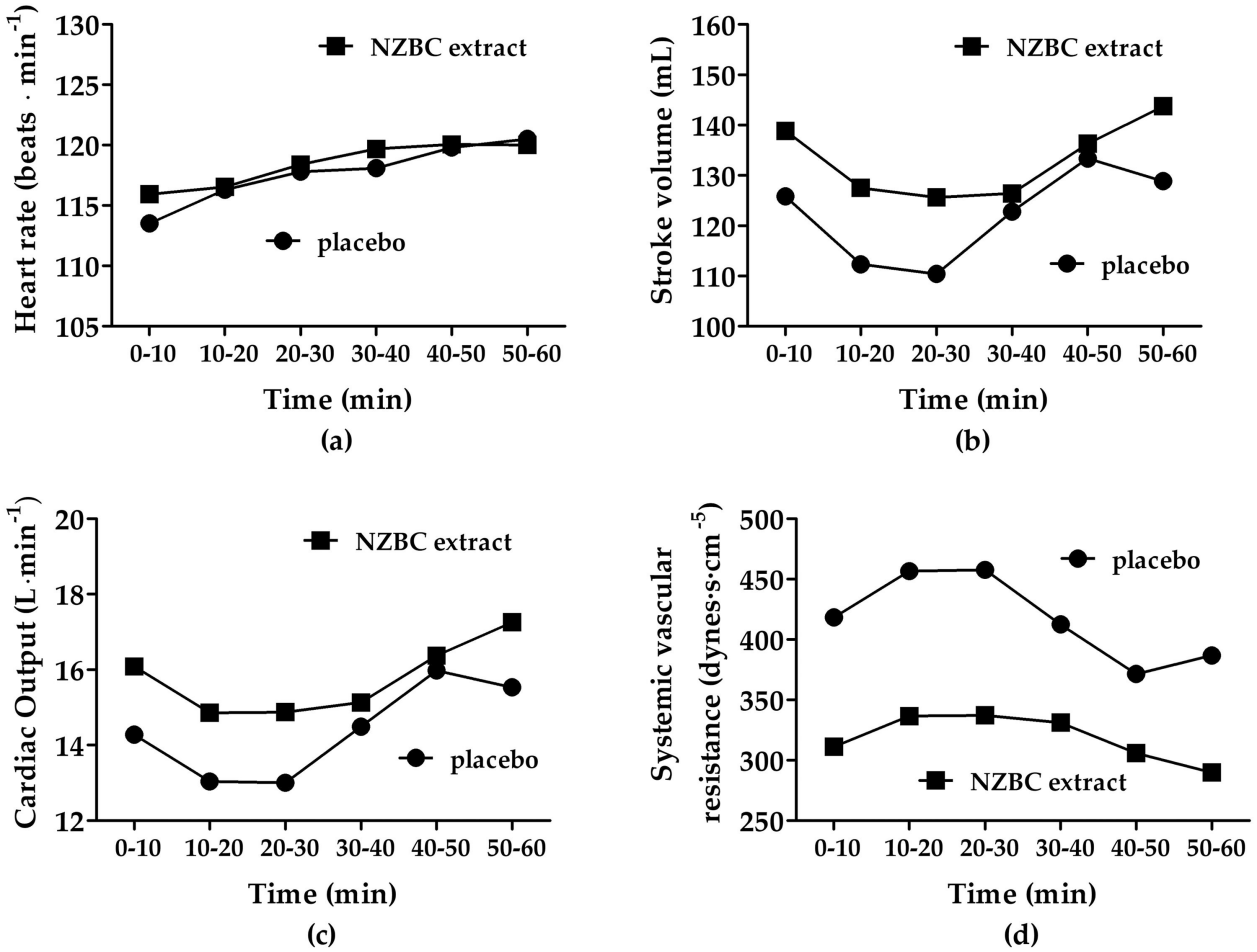
Heart rate **(a)**, stroke volume **(b)**, cardiac output **(c)**, and systemic vascular resistance **(d)** for placebo and New Zealand blackcurrant extract during the 1-h moderate-intensity treadmill run at 50% $\dot{V}O_{2\max}$.

Cardiovascular, metabolic and physiological observations during the 1-h treadmill run at 50%V˙O_2max_ are presented in Table 3. Figure 1 presents the cardiovascular observations during six time periods during the 1-h treadmill run. During the 1-h treadmill run at 50%V˙O_2max_, the New Zealand blackcurrant extract condition was associated with higher stroke volume and cardiac output values, with lower systemic vascular resistance compared with placebo. Heart rate values were similar between conditions [PL: 95% CI (115, 120 beats·min^−1^), NZBC extract: 95% CI (117, 120 beats·min^−1^)], *P* = 014 (Figure 1a). Stroke volume was 9.0% higher in the New Zealand blackcurrant extract condition [PL: 95% CI (113, 132 mL), NZBC extract: 95% CI (125, 141 mL)], *P* < 0.01 (Figure 1b), whereas cardiac output was 9.5% higher [PL: 95% CI (13.09, 15.68 L·min^−1^), NZBC extract: 95% CI (14.74, 16.78 L·min^−1^)], *P* < 0.01 (Figure 1c). Systemic vascular resistance was 23.5% lower in the New Zealand blackcurrant extract condition [PL: 95% CI (380, 454 dynes·s·cm^−5^), NZBC extract: 95% CI (298, 339 dynes·s·cm^−5^)], *P* < 0.001 (Figure 1d).

**Table 3:** Cardiovascular, metabolic and physiological data during the 1-h treadmill run at 50% $\dot{V}O_{2\max}$.

Parameters	Placebo	NZBC extract
heart rate (beats·min^−1^)	118 ± 3	118 ± 2
stroke volume (mL)	122 ± 9	133 ± 8
cardiac output (L·min^−1^)	14.39 ± 1.23	15.76 ± 0.97
SVR (dynes·s·cm^−5^)	417 ± 35	319 ± 19
minute ventilation (L·min^−1^)	36.15 ± 0.23	42.86 ± 1.82
breathing frequency (breaths min^−1^)	36 ± 2	40 ± 2
tidal volume (mL)	1,042 ± 50	1,106 ± 1
oxygen uptake (mL·kg^−1^·min^−1^)	23.12 ± 0.51	24.52 ± 0.40
oxygen uptake (mL·min^−1^)	1,202 ± 75	1,275 ± 21
carbon dioxide production (mL·min^−1^)	940 ± 11	1,031 ± 16
RER	0.78 ± 0.02	0.80 ± 0.02
carbohydrate oxidation (g·min^−1^)	0.42 ± 0.09	0.60 ± 0.10
fat oxidation (g·min^−1^)	0.44 ± 0.04	0.41 ± 0.04

NZBC, New Zealand blackcurrant; SVR, systemic vascular resistance; RER, respiratory exchange ratio. Data for each parameter are presented descriptively as mean ± SD from repeated time intervals during rest or the 1-h treadmill run. Statistical outcomes should be interpreted cautiously because repeated measurements within a single participant do not represent independent observations.

**Figure 2:**
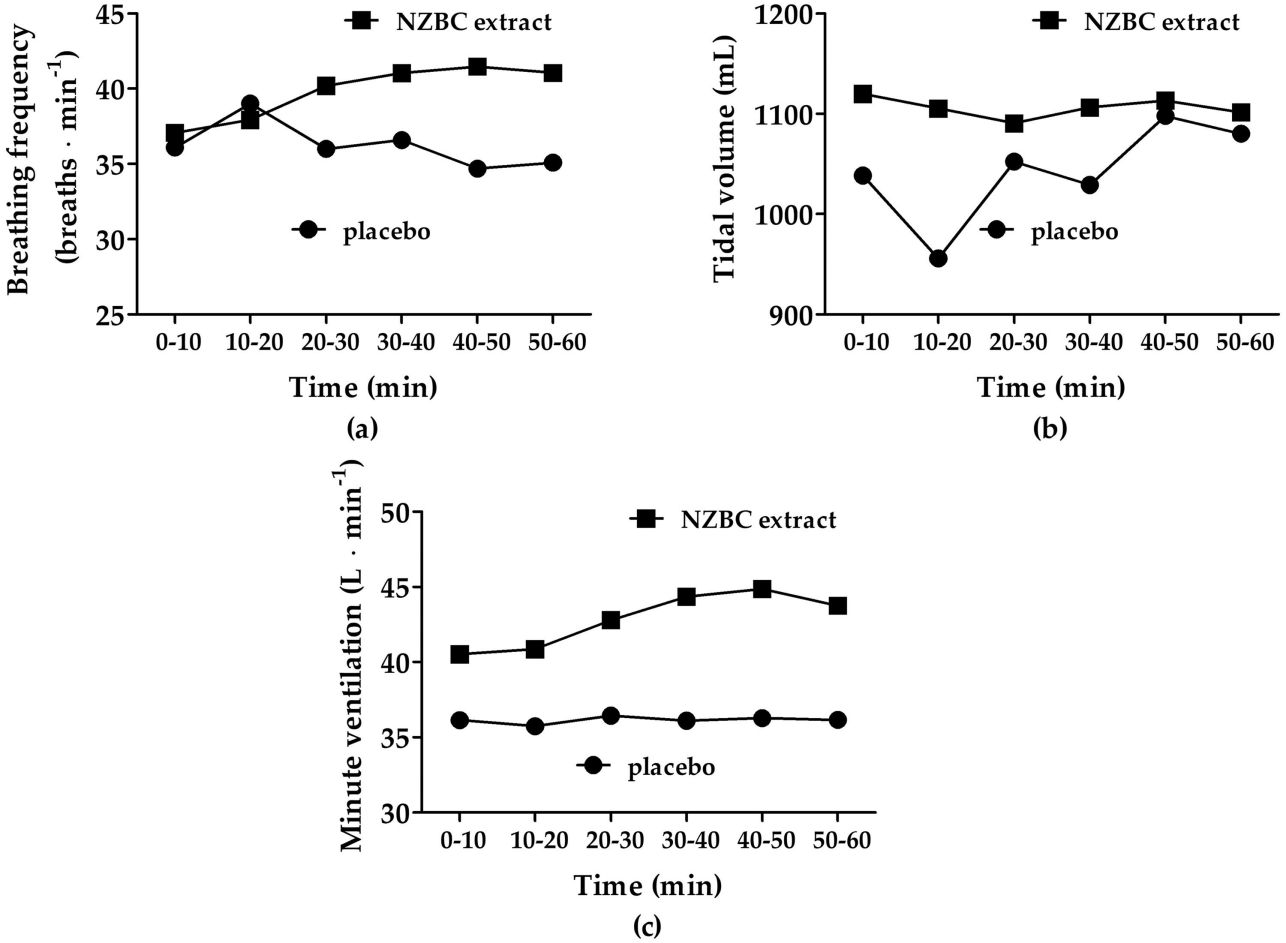
Breathing frequency **(a)**, tidal volume **(b)**, minute ventilation **(c)**, and rating of perceived exertion **(d)** for placebo and New Zealand blackcurrant extract during a 1-h moderate-intensity treadmill run at 50% $\dot{V}O_{2\max}$.

Figure 2 presents the respiratory observations during the six time periods of the 1-h treadmill run. The New Zealand blackcurrant extract condition was associated with higher breathing frequency, tidal volume, and minute ventilation values compared with placebo. Breathing frequency values were 11.1% higher (i.e., 4 breaths·min^−1^) [PL: 95% CI (35, 38 breaths·min^−1^), NZBC extract: 95% CI (38, 42 breaths·min^−1^)], *P* = 0.03 (Figure 2a), tidal volume was 6.1% higher [PL: 95% CI (990, 1,094 mL), NZBC extract: 95% CI (1,096, 1,117 mL)], *P* = 0.04 (Figure 2b), and minute ventilation was 18.6% higher [PL: 95% CI (35.91, 36.39 L·min^−1^), NZBC extract: 95% CI (40.95, 44.77 L·min^−1^)], *P* < 0.01 (Figure 2c). The higher respiratory responses were not accompanied by higher rating of perceived exertion values, which were generally reported as very light to light throughout the treadmill run (Figure 2d).

**Figure 3:**
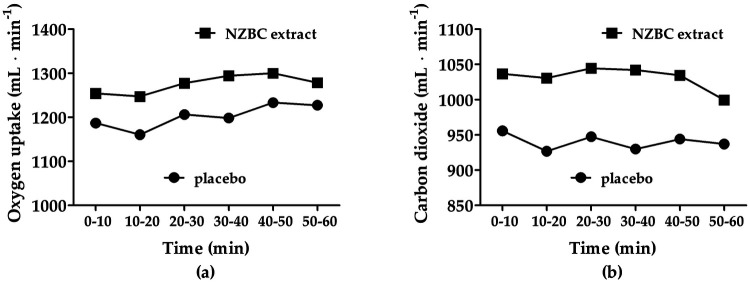
Oxygen uptake **(a)** and carbon dioxide production **(b)** for placebo and New Zealand blackcurrant extract during a 1-h moderate-intensity treadmill run at 50% $\dot{V}O_{2\max}$.

Figure 3 presents the oxygen uptake and carbon dioxide production observations during the 1-h treadmill run. The New Zealand blackcurrant extract condition was associated with higher oxygen uptake and carbon dioxide production values compared with placebo. Oxygen uptake values were 6.1% higher in the New Zealand blackcurrant extract condition [PL: 95% CI (1,174, 1,230 mL·min^−1^), NZBC extract: 95% CI (1,243, 1,297 mL·min^−1^)], *P* < 0.01 (Figure 3a), whereas carbon dioxide production was 9.7% higher [PL: 95% CI (929, 952 mL·min^−1^), NZBC extract: 95% CI (1,014, 1,049 mL·min^−1^)], *P* < 0.01 (Figure 3b).

**Figure 4:**
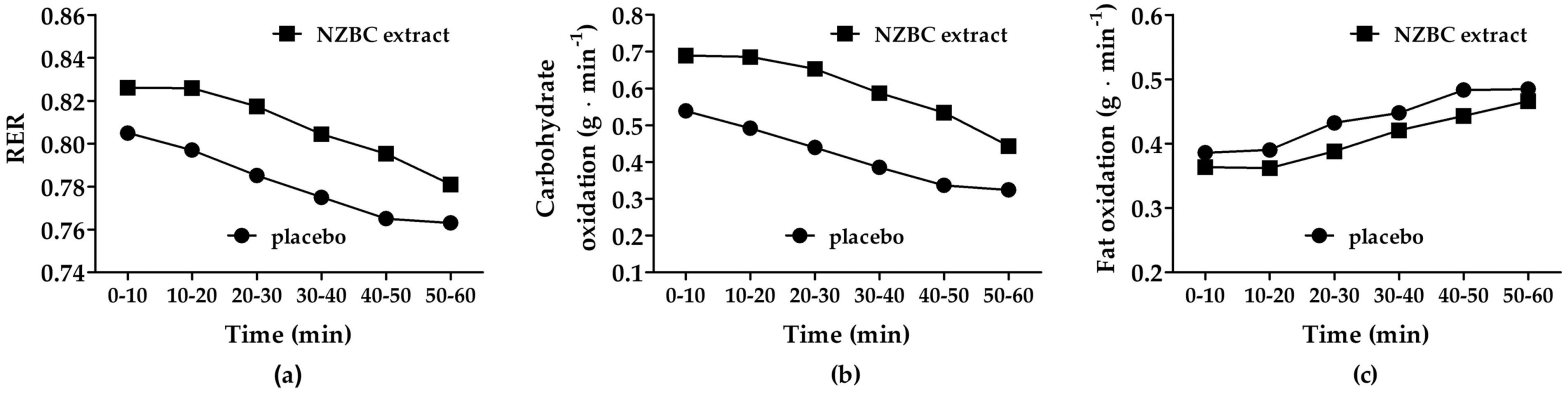
Respiratory exchange ratio **(a)**, carbohydrate oxidation **(b)**, and fat oxidation **(c)** for placebo and New Zealand blackcurrant extract during a 1-h moderate-intensity treadmill run at 50% $\dot{V}O_{2\max}$.

Figure 4 presents the respiratory exchange ratio, carbohydrate oxidation, and fat oxidation observations during the 1-h treadmill run. The respiratory exchange ratio was 0.02 units higher in the New Zealand blackcurrant extract condition [PL: 95% CI (0.76, 0.80), NZBC extract: 95% CI (0.79, 0.83)], *P* < 0.01 (Figure 4a). Carbohydrate oxidation was 42.9% higher in the New Zealand blackcurrant extract condition [PL: 95% CI (0.33, 0.51 g·min^−1^), NZBC extract: 95% CI (0.50, 0.70 g·min^−1^)], *P* < 0.01 (Figure 4b), whereas fat oxidation was 6.8% lower [PL: 95% CI (0.39, 0.48 g·min^−1^), NZBC extract: 95% CI (0.36, 0.45 g·min^−1^)], *P* < 0.01 (Figure 4c).

### 3.3 Lactate 3 min after the 1-h treadmill run

With New Zealand blackcurrant extract, post-run lactate concentration measured 3 min after the 1-h treadmill run at 50%V̇O_2max_ was lower (placebo: 1.7 ± 0.1 mMol·L^−1^; NZBC extract: 0.7 ± 0 mMol·L^−1^). Although the lower post-run lactate concentration represents an interesting physiological observation, it was obtained from a single post-exercise time point and therefore should be interpreted cautiously. Variability related to lactate production, lactate handling, hydration status, substrate availability, running economy, and day-to-day physiological fluctuations cannot be excluded.

The figures are intended to illustrate within-participant temporal response patterns during rest and treadmill running rather than confirmatory inferential effects.

## 4 Discussion

Unique for the present study was 1) the novel dosing strategy that was used for intake of anthocyanin-rich New Zealand blackcurrant extract, 2) the case study participant being an elite National level Southeast Asian female endurance athlete, and 3) that testing occurred 3 weeks before participating in a city marathon (Sydney 2024, Australia). The dosing consisted of 7 days daily intake of 420 mg of blackcurrant anthocyanins using an extract made from New Zealand grown blackcurrants. Most studies on the effects by 7 days intake of New Zealand blackcurrant extract dosed daily with 105 mg [e.g., (11)], and 210 mg [e.g., (17)] of blackcurrant anthocyanins. Only two dose-response studies had as part of the dosing strategy an intake of 315 mg for 7 days (16, 32). The dose of 420 mg of blackcurrant anthocyanins in the present study was selected due to the absence of exercise-induced metabolic changes (i.e., carbohydrate and fat oxidation) but a trend for some cardiovascular changes (i.e., an increase in stroke volume and cardiac output) in Southeast Asian males with 7 days daily intake of 210 mg of blackcurrant anthocyanins (15). These previous observations may suggest that higher anthocyanin dosing strategies could be required in some Southeast Asian individuals to elicit measurable physiological or metabolic responses, although this possibility requires confirmation in adequately powered controlled studies.

In the present study during both supine rest and the moderate-intensity treadmill run at 50% $\dot{V}O_{2\max}$, there were changes for the following parameters. First, cardiac output was higher. The higher cardiac output during supine rest was due to the higher heart rate whereas during the treadmill run, it was due to a higher stroke volume. It is possible that during the treadmill run there was higher return of venous blood with increased cardiac contractility due to the Frank-Starling mechanism (33). In addition, the higher cardiac output both during supine rest and the treadmill run was associated with a lower systemic peripheral resistance indicating vasodilation and enhanced blood flow. The blackcurrant anthocyanins (or the metabolites) may have resulted in vascular regulatory mechanisms that were able to enhance blood flow in humans (18). It is possible that enhanced blood flow contributed to a better clearance of lactate, but this remains speculative, as lower lactate may have due to higher reliance on aerobic energy metabolism. The lower post-run lactate concentration observed with New Zealand blackcurrant extract may reflect altered lactate production, enhanced clearance, differences in oxidative metabolism, or normal day-to-day physiological variability. Therefore, mechanistic explanations regarding nitric oxide activity, endothelial function, or oxidative lactate clearance remain speculative and require confirmation in controlled studies with direct physiological measurements. Lower lactate values of ∼40% were also reported with an acute intake of 420 mg of blackcurrant anthocyanins during 4 h of cycling in an Ironman athlete (26). The lower systemic vascular resistance observed in the present study was associated with increases in oxygen uptake and respiratory responses during the treadmill run. Importantly, the increases in oxygen uptake, respiratory responses, and calculated carbohydrate oxidation during exercise do not necessarily indicate an ergogenic or performance-enhancing effect. At the same running speed and relative exercise intensity, the higher oxygen cost may also reflect altered running economy or substrate utilization characteristics. Therefore, the physiological and performance implications of these responses remain unclear and should be interpreted cautiously. However, there was not a higher perceived exertion during the treadmill run at 50% $\dot{V}O_{2\max}$. Visual inspection of the cardiovascular responses during the treadmill run suggests that stroke volume and cardiac output responses between conditions became more similar during the middle phase of exercise, with greater divergence observed again toward the later stages of the run. Given the exploratory single-case design and the use of averaged time-interval data, these temporal fluctuations should be interpreted cautiously and may reflect normal physiological variability during prolonged steady-state exercise. It is not possible to generalize whether the cardiovascular and respiratory findings during the treadmill run would have beneficial competitive exercise performance effects during endurance activities. It needs to be noted as well that the intensity of the treadmill run (i.e., 50% $\dot{V}O_{2\max}$) was much lower than intensities during competitive marathon running [for a review see (34)]. Future work should examine the effectiveness of intake of New Zealand blackcurrant extract on exercise intensities that are employed during competitive endurance running, as an increase in oxygen uptake is indicative of negatively impacting running economy. It is possible, however, that part of the small increase in oxygen uptake during exercise was due to day-to-day variation and higher breathing frequency in the blackcurrant condition.

In a recent study with a secondary analysis of the individual responses from published studies on the metabolic changes by intake of New Zealand blackcurrant extract (35), it was suggested that individuals with a very low exercise-induced respiratory exchange ratio (RER < 0.80) are more likely to respond with enhanced carbohydrate oxidation. This response pattern differs from the more commonly reported blackcurrant-associated enhancement of fat oxidation observed in previous cohort studies and therefore suggests that physiological responses to anthocyanin-rich supplementation may vary substantially between individuals and exercise contexts. The RER for the female Southeast Asian endurance athlete in the present study was 0.78 during supine rest and during the treadmill run at 50% $\dot{V}O_{2\max}$. The participant demonstrated higher carbohydrate oxidation during rest and treadmill running in the New Zealand blackcurrant extract condition. During the 1-h treadmill run at 50% $\dot{V}O_{2\max}$, the increase of 42.9% for carbohydrate oxidation only amounted to an increase of 10.8 g of carbohydrate oxidation. Therefore, it is unlikely that the enhanced carbohydrate oxidation by intake of New Zealand blackcurrant extract in individuals with a very low exercise-induced respiratory exchange ratio would require to alter substantially the carbohydrate dosing strategies during endurance activities. The mechanisms underlying the observed alterations in carbohydrate oxidation remain unclear. Potential explanations may include modulation of oxidative metabolism or effects on glucose handling pathways. However, no direct biochemical, molecular, or metabolic pathway measurements were obtained in the present study. Therefore, mechanistic interpretations regarding antioxidant-mediated effects on glycolytic enzyme activity, glycogen phosphorylase activation, or glucose transporter regulation remain speculative and require confirmation in controlled mechanistic investigations. Previous animal research has reported enhanced skeletal muscle glucose transporter 4 expression following anthocyanin-rich supplementation (36), but whether similar responses occur in humans during exercise remains unknown.

Finally, it needs to be emphasized that the participant in the present case study was a heat-acclimatized, highly endurance-trained elite National Southeast Asian female athlete living and training in Thailand. Environmental conditions, habitual diet, and endurance training status may influence physiological responses to blackcurrant anthocyanin supplementation. However, gut microbiome composition, anthocyanin metabolite availability, and related physiological adaptations were not measured in the present study; therefore, any mechanistic interpretation involving microbiome adaptation remains speculative. In addition, observations from single-participant case studies are low in the hierarchy of scientific evidence [e.g., (37)], and caution is required for generalization of the observations. Further caution is required as the repeated measurements of the physiological, metabolic and cardiovascular parameters at time intervals in the placebo and blackcurrant condition and subsequent analysis with paired *t*-test may be considered pseudo-replication. A limitation of the study was the absence of dietary standardization in the days before testing and testing for the placebo condition first potentially inducing an order effect on the observations. Although the participant was instructed to replicate dietary intake before each experimental visit and dietary records were reviewed for general consistency, effects of differences in carbohydrate intake or glycemic availability between conditions cannot be excluded. We did not record menstrual cycle information as female-specific physiological factors such as menopausal status, menstrual cycle characteristics, hormonal fluctuations, and endocrine status. Finally, we cannot exclude that our observations were affected by the participant being in a tapering phase before marathon competition.

The present study has several important limitations. As a single-participant case study without randomization or counterbalancing, the findings are exploratory and hypothesis-generating rather than confirmatory. In addition, the placebo condition preceded the blackcurrant extract condition; therefore, potential order effects, learning effects, training adaptations, recovery status, or taper-related physiological changes before marathon competition cannot be excluded and may have contributed to the observed responses. Day-to-day physiological variability of cardiovascular, physiological and metabolic responses may also have influenced the findings. Therefore, causal inferences regarding blackcurrant extract supplementation should be avoided, and the observations should not be generalized beyond the present athlete. The participant's age may also have influenced cardiovascular, metabolic and physiological responses and should be considered in future investigations involving female endurance athletes. Moreover, blood glucose was not measured before or during the treadmill-running trials; therefore, differences in pre-exercise or exercise-related glycemic status cannot be ruled out. Furthermore, coefficient of variation data for lactate measurements were not available in the present study, which limits interpretation of the post-run lactate observations.

## 5 Conclusion

It was concluded that seven days of daily intake of an extract of New Zealand blackcurrant with 420 mg of anthocyanins was associated with alterations in metabolic, physiological, and cardiovascular responses during supine rest and moderate-intensity treadmill running in one elite National Southeast Asian female endurance athlete living and training in Thailand. The cardiovascular responses may suggest systemic vasodilation. Given the exploratory single-case design without randomization or counterbalancing, the findings should be interpreted cautiously and cannot be generalized beyond the present participant and future well-controlled cohort studies are required to determine whether similar responses occur in larger cohorts of female endurance athletes. Future studies should also address whether the dosing strategy with 420 mg of blackcurrant anthocyanins can provide beneficial effects for competitive exercise endurance performance.

## Data Availability

The raw data supporting the conclusions of this article will be made available by the authors, without undue reservation.
